# Effect of Initial Fraction of Cooperators on Cooperative Behavior in Evolutionary Prisoner's Dilemma Game

**DOI:** 10.1371/journal.pone.0076942

**Published:** 2013-11-07

**Authors:** Keizo Shigaki, Zhen Wang, Jun Tanimoto, Eriko Fukuda

**Affiliations:** 1 Interdisciplinary Graduate School of Engineering Sciences, Kyushu University, Kasuga-koen, Kasuga-shi, Fukuoka, Japan; 2 Department of Physics, Hong Kong Baptist University, Kowloon Tong, Hong Kong; 3 Center for Nonlinear Studies and the Beijing-Hong Kong-Singapore Joint Center for Nonlinear and Complex systems (Hong Kong), Hong Kong Baptist University, Kowloon Tong, Hong Kong; University of Zaragoza, Spain

## Abstract

We investigate the influence of initial fraction of cooperators on the evolution of cooperation in spatial prisoner's dilemma games. Compared with the results of heterogeneous networks, we find that there is a relatively low initial fraction of cooperators to guarantee higher equilibrium cooperative level. While this interesting phenomenon is contrary to the commonly shared knowledge that higher initial fraction of cooperators can provide better environment for the evolution of cooperation. To support our outcome, we explore the time courses of cooperation and find that the whole course can be divided into two sequent stages: enduring (END) and expanding (EXP) periods. At the end of END period, thought there is a limited number of cooperator clusters left for the case of low initial setup, these clusters can smoothly expand to hold the whole system in the EXP period. However, for high initial fraction of cooperators, superfluous cooperator clusters hinder their effective expansion, which induces many remaining defectors surrounding the cooperator clusters. Moreover, through intensive analysis, we also demonstrate that when the tendency of three cooperation cluster characteristics (cluster size, cluster number and cluster shape) are consistent within END and EXP periods, the state that maximizes cooperation can be favored.

## Introduction

One major challenge in fields ranging from genetics and cell biology to evolutionary anthropology and behavioral economics is the emergence and persistence of cooperation [1], [2]. In order to understand this trait, evolutionary game theory, that sheds light into long-time issue, has been proved to be a very useful tool [3], [4]. In particular, a simple and paradigmatic model, the prisoner's dilemma game (PD), has attracted considerable attention from theoretical and experimental viewpoints [5]–[12]. In its basic version, two players simultaneously decide to adopt one of strategies: cooperation (C) or defection (D). If both cooperate (defect) they receive the reward *R* (the punishment *P*). If, however, one player chooses cooperation while the other defects, the latter gets the temptation *T* and the former is left with the sucker's payoff *S*. These payoffs satisfy the ranking *T*>*R*>*P*>*S* and 2*R*>*T*+*S*; thus, defection optimizes the individual payoff, in spite of the fact that mutual cooperation could yield a higher collective benefit. Resulting is a social dilemma, which typically leads to widespread defection. To overcome this unfortunate outcome, special scenarios that support the evolution of cooperation need to be suggested.

Over the past decades, a great number of mechanisms has been identified that can offset an unfavorable outcome of social dilemmas and lead to the evolution of cooperation [13]–[16]. Typical examples include voluntary participation in social interactions [17]–[19], spatially structured populations [20]–[25], the mobility of players [26]–[30], heterogeneous ability and aspiration [31]–[35], co-evolutionary selection of dynamical rules [36]–[39] and so on. Whereas, Nowak attributed all these to five mechanisms: kin selection, direct reciprocity, indirect reciprocity, network reciprocity, and group selection [40], these mechanisms can be somewhat related to the reduction of an opposing player's anonymity. Among the five mechanisms, network reciprocity, where players are arranged on the spatially structured topology and interact only with their direct neighbors, has attracted the greatest interest (for comprehensive reviews refer to Refs. [41],[42]), because cooperators can survive by means of forming compact clusters, which minimize the exploitation by defectors and protect those cooperators that are located in the interior of such clusters [43]. In line with the research of Nowak and May [43], more complex spatial topology has been investigated to extend the scope of cooperation on interaction network [44]–[53]. For example, in a recent research work [54], where heterogeneous scale-free network was employed as the potential interaction topology, the state of fully cooperative dominance was reported. It was shown that a high value of the clustering coefficient was beneficial for the evolution of cooperative traits, and could lead to the long-term sustenance of cooperation [55],[56]. On the other hand, the increasing body of knowledge on human interaction arising from experimental economics has also identified the effective role of spatial topology in promoting cooperation [57].

In spite of relative large body of work that has been accumulated, there is a situation of particular relevance that has received relatively little attention till now. That is the impact of the initial fraction of cooperators on the final equilibrium state. In a recent research [58], where the stochastic updating rule and heterogeneous networks were implemented, it was demonstrated that the characterization of the asymptotic states of the evolutionary dynamics was largely independent of the initial concentration of cooperators. However, it was obvious that the stochastic influence coming from updating rule and heterogeneous topology always existed within such a framework. Based on the achievement, one question poses itself, which we aim to address in what follows. Namely, if we remove these stochastic factors, how does the initial concentration of cooperators affects the final equilibrium states?

In the present work, we use the deterministic updating rule and homogeneous interaction networks to explore the potential effect brought by the initial fraction of cooperators. Through systematic study, we derive the time course of cooperation evolution into two sequential processes: enduring (END) and expanding (EXP) periods. Of particular interest, we inspect that the initial fraction of cooperators directly affects the final level of cooperation on the interaction topology.

## Spatial Model of Prisoner's Dilemma

We consider the evolutionary prisoner's dilemma game with players located on the sites of the network. Each player *i* can adopt one of the two strategies: cooperation (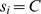) or defection (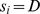). For simplicity, but without loss of generality, we use a standard parameterization of the game: reward for mutual cooperation *R* = 1, punishment for mutual defection *P* = 0, then the payoff matrix can be rescaled as follows,
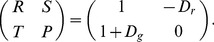
(1)where *D_r_* = *P*−*S* (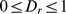) is the so-called stag-hunt-type dilemma and *D_g_* = *T*−*R* (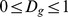) denotes the chicken-type dilemma [59],[60]. In the present study, we focus on PD games by limiting 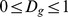, 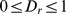 and *r* = 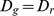, where *r* is the cost-to-benefit ratio [61].

The game is iterated forward in accordance with the sequential simulation procedure comprising the following elementary steps. First, each player *i* acquires its payoff *P_i_* by playing the game with all its neighbors. Then players update their strategies synchronously. Here, we mainly pay attention to the deterministic rule: Imitation Max (IM). The Imitation Max (IM) policy can be described in the following way: the strategy 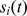 of individual *i* at time step *t* will be
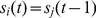
(2)where *j* is one member among player *i* and all her neighbors 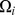 (namely, 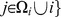 *j*∈{*Vi*∪*i*}) such that 
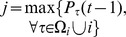
. Notably, the selection of IM rule can also get the interpretation in different fields. From a purely economic viewpoint, entities are more inclined to imitate the policy of companies gaining highest benefit. While in the social systems, the behavior of most successful agents is always adopted by their followers.

As the interaction network, we mainly use the homogeneous cycle and square lattice with periodic boundary conditions and 4 or 8 nearest neighbors. To further validate the possible influence of stochastic factor, heterogeneous networks: regular random graph (RRG) and the scale-free (SF) network with average degree of 4 or 8 (i.e., <*k*> = 4 or 8) are also considered. The total number of agents is *N* = 10000.

## Enduring and Expanding Periods

**Figure 1:**
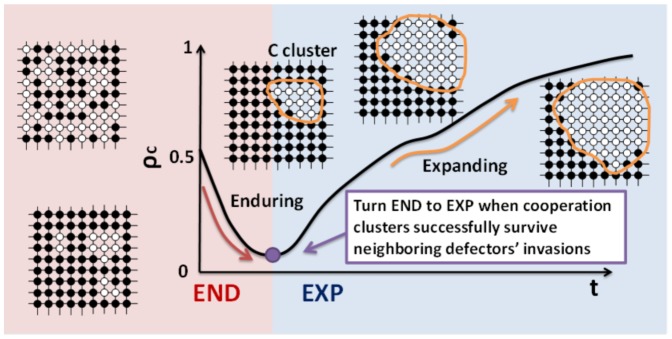
Schematic view for the evolution of cooperation in spatial prisoner's dilemma game with concept of END and EXP. Enduring (END) period: Initial cooperators will be rapidly plundered by defectors, which cause only few cooperators left through forming compact C-clusters. Expanding (EXP) period: C-clusters start to expand, since a cooperator on the clusters' border can attract a neighboring defector into the cluster.

For the sake of the following discussion, we define the terminology as the enduring (END) period and the expanding (EXP) period as shown in Fig. 1. In a typical evolution course, where the initial value of the cooperation fraction is 0.5, there are usually two evident processes: the former period features the rapid downfall of cooperation, while the following one is along with the increase of cooperation level unless the evolutionary trail is absorbed by all-defectors state during the foregoing period. In our study the first is the so-called enduring (END) period because cooperators try to endure defectors' invasion (or cooperators avoid learning defection from neighbors). Correspondingly, we call the other the expanding (EXP) period, since cooperators, who successfully survive in the END period by forming cooperative clusters (C-clusters), expand their area by converting defectors into cooperators. In some particular cases (such as, weak dilemma), when the initial fraction of cooperation is low, the evolutionary trail may start from EXP period.

## Cluster Characteristics

To feature how emerging spatial patterns qualitatively affect the evolution of cooperation, three cluster characteristics: cluster number *N_C_*, cluster size *S_C_* and cluster shape *SH_C_* of cooperator aggregations are employed [50]. In particular, we need to give the detailed definition of the third term. For each cluster *i*, we can derive *SH_Ci_* based on the number of C-C links, *l_CC_*, within cluster *i* and the number of C-D links, *O_CD_*, that connect cluster *i* with the surrounding defectors:
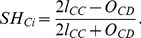
(3)The value of *SH_Ci_* is constrained to the interval [−1, 1]. Obviously, compact C-cluster has more links within the cluster rather than to the surrounding defectors. The value of *SH_Ci_* is positive, which indicates positive assortment of cooperators. While for the sparse cluster there are fewer links within the cluster but more links connecting to surrounding defectors. Thus, *SH_Ci_*<0 and negative assortment among cooperators takes place (or positive assortment between cooperators and defectors). Moreover, in order to eliminate the influence of isolated cooperators, the cluster size *S_C_* and cluster shape *SH_C_* are weighed such that the weight of each cluster corresponds to its size.

## Results and Discussion

We have performed extensive numerical simulations under different interaction networks. The equilibrium fraction of cooperation 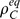 is determined within the last 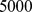 generations out of the total 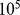 iteration steps. Moreover, to guarantee validity and statistical robustness of data, the final results are averaged over up to 100 independent runs for each set of parameter values. During one time step, the agents update their strategies synchronously. In all the figs, we use 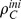 to denote the initial fraction of cooperators.

### (A). Effect of initial fraction of cooperators

**Figure 2:**
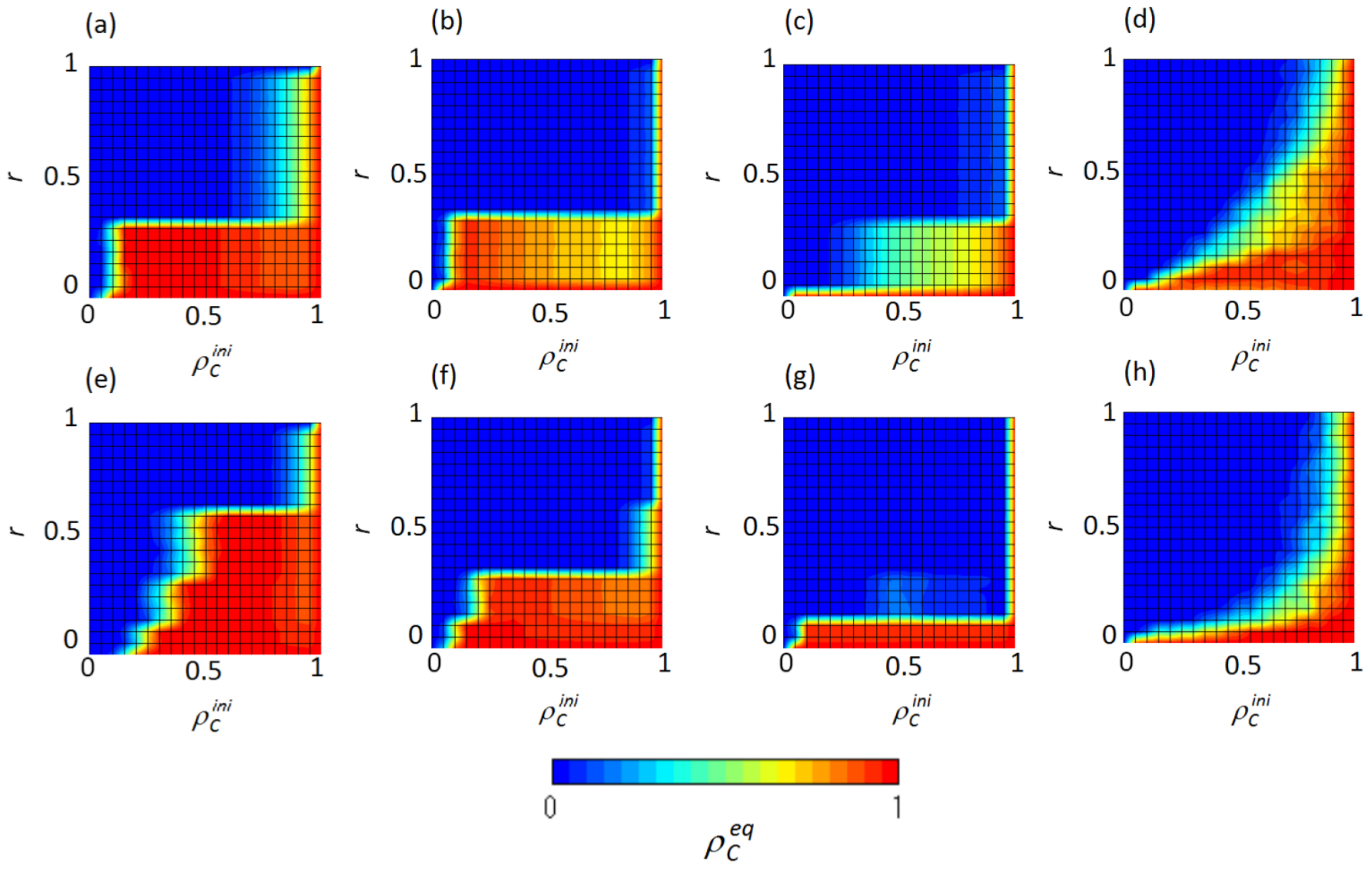
Equilibrium fraction of cooperators 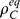 on the 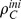-*r* parameter plane on various topologies. From left to right ((a)–(d) and (e)–(h)), the interaction networks are cycle network, square lattice network, RRG and the scale-free network, respectively. For top panel, the average degree is <*k*> = 4, for bottom panel it is <*k*> = 8.

We start by presenting the color map encoding the equilibrium fraction of cooperators 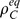 on the 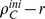 parameter plane for different interaction topology networks and degree in Figure 2. It is noteworthy that, on homogeneous network, higher initial fraction of cooperators 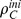 does not necessarily lead to the high equilibrium fraction of cooperation 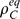 in weaker dilemma region (*r*<0.3), especially on the square lattice with low degree (see Fig. 2 (b)). On the contrary, a relatively low initial fraction of cooperators can induce the undisputed dominance of cooperation. While for the heterogeneous networks, a higher initial fraction of cooperators usually provides better environment for the evolution of cooperation. This can be easily explained by the fact that enhanced initial fraction of cooperators increases the possibility of cooperators holding the highly connected nodes (such as, hub nodes), which in turn attract their neighbors into cooperators and guarantee in this way their long-time success [58]. Thus, these results suggest that when the stochastic factor is removed (namely, the combination of deterministic updating rule and homogeneous interaction topology), a relatively low initial fraction of cooperators can create better environment for the sustenance of cooperation. To simplify the discussion, we mainly focus on the case of square lattice with *k* = 4.

### (B).Square lattice network (*k* = 4, IM)

**Figure 3:**
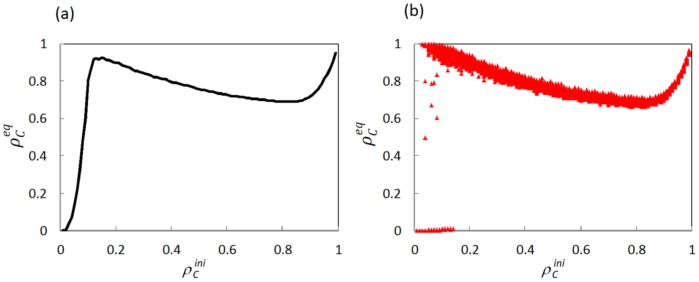
Equilibrium fraction of cooperators 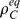 in dependence on the density of initial cooperators 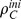 on the square lattice network with *k* = 4 and IM rule. Panel (a) illustrates the average fraction of cooperators, while (b) corresponds to the level of cooperation reached in each of the 100 realizations. Depicted results are obtained for *r* = 0.2.

Figure 3 shows how 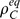 varies as a function of the initial fraction of cooperators 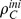 on a square lattice with *k* = 4 and IM rule. It is clear that the region of 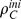
≈0.15 exhibits higher cooperation level than the other regions with a relatively low or high initial fraction of cooperators, which, to some extent, is similar to phenomenon of evolutionary resonance [62]. For lower initial fraction of cooperators (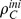
<0.15), though few cases can reach the coexistence phase of cooperators and defectors, majority of the realizations is absorbed by the phase of pure defectors. On the other hand, a relatively high initial fraction of cooperators (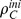
>0.15) shows that its dynamics always ends up with the coexisting equilibrium in which cooperators and defectors simultaneously survive. To explain these phenomena, we quantitatively explore the characters of microscopic evolution dynamics.

**Figure 4:**
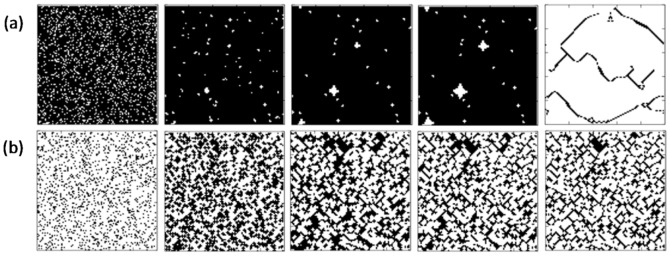
Evolutionary snapshots for cooperators (white) and defectors (black) under different initial fraction of cooperators: (a) 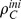 = 0.15 and (b) 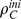 = 0.85. Form left to right, the snapshots are given at t = 0, 1, 3, 5, 100 steps for all panels. Depicted results are obtained for *r* = 0.2.

Figure 4 presents characteristic evolution snapshots of cooperators and defectors for different initial fractions of cooperators; (a) 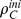 = 0.15 and (b) 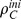 = 0.85, respectively. In the case of low initial cooperation fraction, the system rapidly falls into the state of numerous defectors. However, at the end of EXP period, a few C-clusters can successfully survive under the exploitation of free-riders, and then these remaining C-clusters start recovering lost ground against weakened defectors. During the END period, a defector is always ready to change its status and tries to penetrate into the C-clusters, which finally yields the exclusive dominance of cooperators. On the other hand, for large initial value of 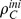, a great number of C-clusters can survive during the END period. But these C-clusters mutually hinder the expansion of others in EXP period (namely, can not expand enough smoothly), which thus induces many remaining defectors surrounding the C-clusters.

**Figure 5:**
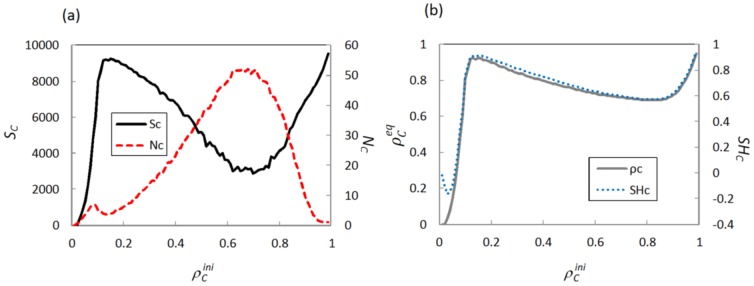
Macroscopic features for the cluster characteristics and the equilibrium fraction of cooperators. Panel (a): cluster size *S_C_* and cluster number *N_C_* as well as (b): equilibrium fraction of cooperators 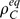 and cluster shape *SH_C_* in dependence on the initial fraction of cooperators 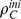. Depicted results are obtained for *r* = 0.2.

**Figure 6:**
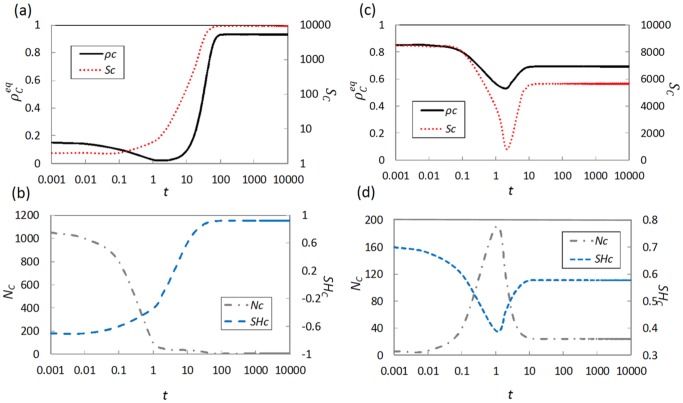
Time evolution of the fraction of cooperators (top panels, black solid line), cluster size (top panels, red dotted line), cluster number (bottom panels, gray dashed-dotted line) and cluster shape (bottom panels, blue dashed line) for 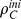 = 0.15 (left) and 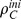 = 0.85 (right) when assuming *r* = 0.2. Each of the time evolution curves indicates an ensemble average of 100 realizations.

Furthermore, we quantify the effect of 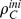 from the viewpoint of cluster characteristics. Figure 5 features how three C-cluster characteristics and the equilibrium fraction of cooperators change versus initial fraction of cooperators. In the region of 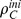
≈0.15, where the cooperation reaches the optimal state, both cluster size *S_C_* and cluster shape *SH_C_* also obtain their maximum values. On the contrary, cluster number *N* *_C_* shows its minimum value. This means that the formed C-clusters in the END period can most effectively expand and even dominate the whole system. In order to further support our results, we examine the time course for fraction of cooperators and three cluster characteristics under different initial fractions of cooperators in Figure 6. For 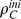 = 0.15, during both END and EXP periods, the cluster size *S_C_* and the cluster shape *SH_C_* monotonically increase, while the cluster number *N_C_* decreases (see Fig. 6 (a), (b)). Correspondingly, in the case of 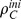 = 0.85, the cluster size *S_C_* and the cluster shape *SH_C_* decrease, while the cluster number *N_C_* increases during the END period, However, concerned with the EXP period, the cluster size *S_C_* and the cluster shape *SH_C_* start to increase, the cluster number *N* *_C_* decreases (see Table 1). It is interesting that the change of evolution trend for cluster characteristics prevents the smooth expansion of C-clusters in EXP period. Thus, the consistency of trend for three cluster characteristics during END and EXP periods may be seen as an effective index to estimate whether the initial state is able to bring a highly cooperation equilibrium.

**Table 1:** Cluster characteristics (cluster size, cluster number and cluster shape) for two different initial fractions of cooperators 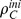 = 0.15, 0.85.

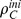 = 0.15	END	EXP	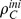 = 0.85	END	EXP
*S_C_*	increase	increase	*S_C_*	decrease	increase
*N_C_*	decrease	decrease	*N_C_*	increase	decrease
*SH_C_*	increase	increase	*SH_C_*	decrease	increase

Note that in the case of 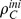 = 0.15, the tendency of three cluster characteristics is the same from END to EXP periods, on the other hand, in the case of 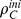 = 0.85, the tendency of three cluster characteristics is different within END and EXP periods.

## Conclusion

We have investigated the effect of initial fraction of cooperators on the equilibrium level of cooperation. Our results show that when homogeneous network is assumed as the underling topology, an interesting phenomenon takes place: relatively low initial fraction of cooperators can lead to a higher level of cooperation at equilibrium. To support this, we examine the time courses. We find that for low initial fraction of cooperators, a few C-clusters can survive during the END period. And, in the following EXP period, these C-clusters expand smoothly till they dominate nearly the whole system. On the other hand, when one starts from a high initial fraction of cooperators, a great number of C-clusters survive during END period. But these C-clusters can not guarantee the prosperity of cooperation because they mutually hinder the expansion of other clusters in the EXP period. Moreover, we study the performance of three cluster characteristics: cluster size, cluster number and cluster shape under different initial cooperation setup. If one observes coherent tendency of those three cluster characteristics in both END and EXP periods, the scope of cooperation could be extended.
